# Supplementary material for: Identification of HuSWEET Family in Pitaya (Hylocereus undatus) and Key Roles of HuSWEET12a and HuSWEET13d in Sugar Accumulation
Source: Int J Mol Sci. 2023 Aug 17;24(16):12882. doi: 10.3390/ijms241612882 (PMC10454816; doi:10.3390/ijms241612882)
Supplement: Supplementary file 1 [file ijms-24-12882-s001.zip › Figure S1.pdf]

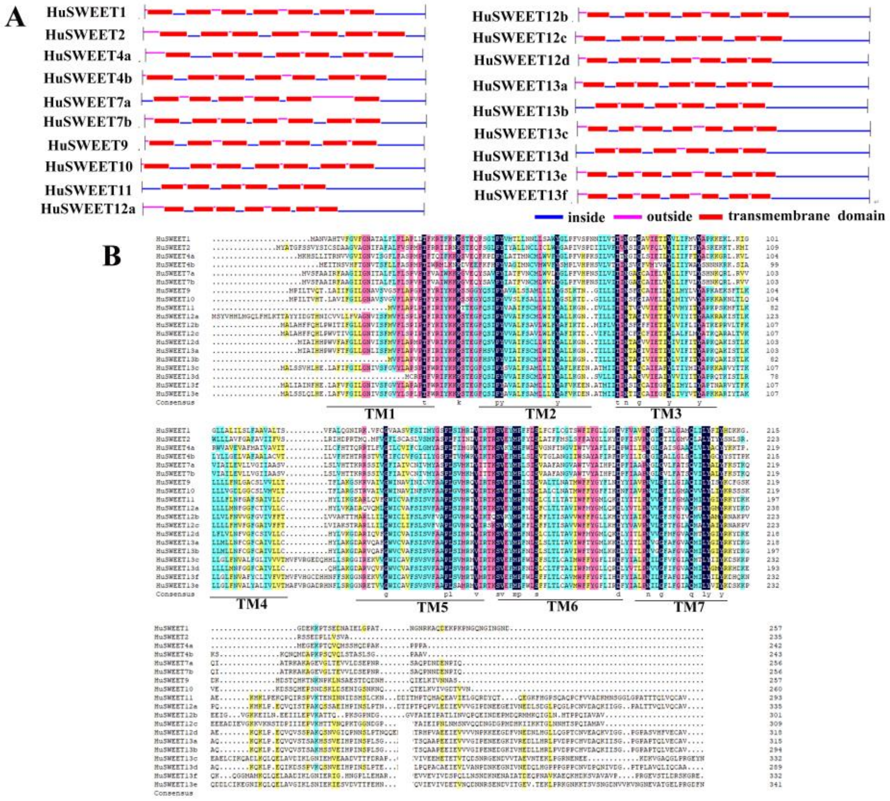

Figure S1. Multiple sequence alignments and transmembrane (TM) domains of HuSWEETs (A) The positions of the seven TMs of the pitaya. (B) Multiple sequence alignments of HuSWEETs. The positions of the seven TMs are indicated by lines.
